# The prognostic influence of tumor infiltrating Foxp3^+^CD4^+^, CD4^+^ and CD8^+^ T cells in resected non-small cell lung cancer

**DOI:** 10.1186/s12950-015-0108-x

**Published:** 2015-11-23

**Authors:** Jurgita Jackute, Marius Zemaitis, Darius Pranys, Brigita Sitkauskiene, Skaidrius Miliauskas, Vytis Bajoriunas, Simona Lavinskiene, Raimundas Sakalauskas

**Affiliations:** Department of Pulmonology and Immunology, Medical Academy, Lithuanian University of Health Sciences, Kaunas, Lithuania; Department of Pathology, Hospital of Lithuanian University of Health Sciences, Kaunas, Lithuania; Department of Heart, Thoracic and Vascular Surgery, Medical Academy, Lithuanian University of Health Sciences, Kaunas, Lithuania

**Keywords:** Non-small cell lung cancer, Foxp3 + CD4+ T cells, CD4+ T cells, CD8+ T cells, Tumor islets and stroma, Immunohistochemistry, Smoking

## Abstract

**Background:**

Different subsets of tumor infiltrating T lymphocytes are believed to play essential role in the immune response to cancer cells. The data of these cells in NSCLC are relatively rare and controversial therefore we aimed to evaluate the infiltration patterns of Foxp3 + CD4+, CD4+ and CD8+ T cells in NSCLC and to analyze their relations to survival.

**Methods:**

Lung tissue specimens from 80 newly diagnosed and untreated patients who underwent surgery for NSCLC (stages I-III), and 16 control group subjects, who underwent surgery due to recurrent spontaneous pneumothorax, were analyzed. Foxp3 + CD4+, CD4+ and CD8+ T cells in tumor stroma and islets were evaluated immunohistochemically. All statistical analyses were performed using the Statistical Package for the Social Sciences (SPSS), version 20.0.

**Results:**

Tumor infiltrating CD4+, CD8+ T cells were associated with neither overall survival nor disease-free survival. The presence of high tumor stroma infiltrating Foxp3 + CD4+ T cells was independently associated with improved NSCLC patients overall survival (*P* < 0.05).

**Conclusions:**

Our study demonstrated that tumor infiltrating Foxp3 + CD4+ T cells are associated with improved NSCLC patients' survival. In addition our findings highlight a tendency of high CD4+/CD8+ and CD8+/Foxp3 + CD4+ T cells ratio in prolonged NSCLC patients' survival.

## Introduction

Lung cancer is most frequent carcinoma and leading cause of cancer-related mortality. Immune responses within the tumor microenvironment are increasingly implicated as determining factor in tumor progression and aggressiveness. Understanding these responses has allowed investigators to use immune markers to stratify the prognosis of NSCLC patients [1]. Tumor-associated immune response is pivotal factor in tumor progression. Tumor infiltrating T lymphocytes play an important role in modeling antitumor immune response. As the survival of NSCLC remains very poor despite the newest anticancer treatment, prognostic markers are required to distinguish the patients with poor and good prognosis in order to choose the better treatment and follow-up strategy. Different subsets of tumor infiltrating T lymphocytes are believed to play essential role in the immune response to cancer cells.

However, tumor progression, which often also is seen in the presence of substantial lymphocytic infiltration, suggests that T cells are not capable provide effective immune responses to control tumor growth [2]. Evidence has accumulated that tumor infiltrating T lymphocytes are functionally defective, incompletely activated, and include regulatory subtypes, which varied with the types of cancers [3–5]. Therefore, if distinctions were made with respect to lymphocyte types, location, functional state, and their interactions, a more profound impact on prognosis was observed compared with only the overall degree of lymphoid infiltration [6]. Recently Foxp3^+^CD4^+^ T cells became an object of great interest in studies of different cancer types as well as in NSCLC. It is thought that Foxp3^+^CD4^+^ T cells have the ability to suppress or regulate cell-mediate immunity [7]. After migrating to tumor site, Foxp3^+^CD4^+^ T cells can inhibit the activity of cytotoxic T cells through cell-to-cell contact. However different mechanisms by which Foxp3^+^CD4^+^ T cells suppress antitumor immune response exist, but still not fully understood yet [8]. Although Foxp3^+^CD4^+^ T cells can potentially promote cancer progression, they can also attenuate inflammation. Because chronic inflammation is one of the critical processes promoting carcinogenesis and tumor growth, Foxp3^+^CD4^+^ T cells are able to down-regulate the pro-tumorigenic inflammatory responses.

Some Foxp3^+^CD4^+^ T cells associated molecules are involved in carcinogenesis process such as IL-10 and TGFβ [9]. IL-10 is related with impaired antigen presentation for proper activation of T cells and T cell proliferation; TGFβ may suppress natural killer cell function. Hatanaka et al. [10] found that NSCLC patients have elevated IL-10 serum levels compared to healthy controls which has been associated with poorer prognosis. Nevertheless, data concerning the role of IL-10 for tumor progression are partially inconsistent. In contrast to these results, some studies suggest that IL-10 is important for tumor rejection [11, 12]. Despite these contradictions, the immune-suppressive cytokine is an important factor during the induction of airway tolerance. Therefore, it is necessary to further define the role of IL-10 in NSCLC [9].

Tumor-infiltrating CD4^+^ and CD8^+^ T cells play important role in the immune response to cancer cells in NSCLC. As CD8^+^ T cells exhibit marked cytotoxic capacities that may induce tumor cell death [13], by releasing perforins and granzymes in acquired immune responses, thereby playing a critical role in antitumor immunity [14]. Indeed, CD8^+^ T cells are most likely functionally relevant in NSCLC, as the number of apoptotic tumor cells was significantly higher in tumors with a high number of CD3^+^ and CD8^+^ T cells [15] therefore CD8^+^ T lymphocytes comprise a well-established group of effector T cells with potent cytotoxic effects in cancer [16]. However, the presence of CD8^+^ T cells in NSCLC has been identified as positive, neutral or negative prognostic marker [17–19]. Another important factor affecting clinical outcomes in NSCLC might be tumor infiltrating CD4^+^ T cells. These cells produce immunoregulatory cytokines such IFN-gamma and TNF that may induce cytolytic CD8^+^ T cell responses in lung cancer [14, 20]. Another important factor effecting clinical outcomes in NSCLC might be relatinship between CD4^+^ and CD8^+^ T cells due to synergistic compartment [20].

The data of these cells in NSCLC are relatively rare and controversial therefore we aimed to evaluate the infiltration patterns of Foxp3^+^CD4^+^, CD4^+^ and CD8^+^ T cells in tumor islets and stroma from patients with NSCLC and to analyze their relations to survival.

## Material and methods

### Subjects

Lung tissue specimens from 80 newly diagnosed and untreated NSCLC patients who underwent surgical resection for NSCLC (pathological stage I-III) and 16 control group subjects who underwent surgery due to recurrent spontaneous pneumothorax at the Hospital of Lithuanian University of Health Sciences, Kaunas Clinics from September 2012 to April 2015 were studied. For eligible patients, demographic data including smoking habit, data on COPD and other comorbidities were collected. Subjects were excluded if they had a history of another malignancy, connective tissue diseases or any unstable systemic disease (including active infections, significant cardiovascular disease). Peripheral venous blood samples were obtained before lung surgery. None of NSCLC patients received preoperative radiotherapy or chemotherapy. Two qualified pathologists evaluated lung specimens. Histological classification of tumors was based on the World Health Organization criteria [21]. Tumors were staged according to the TNM Classification of Malignant Tumors, the seventh edition [22]. The clinical stage, tumor type were recorded at the time of diagnosis. NSCLC patients were divided into two groups according to their COPD status: NSCLC patients with COPD and NSCLC patients without COPD. COPD was defined according to the criteria of the Global Initiative for Chronic Obstructive Lung Disease (GOLD) [23]. None of included subjects had an exacerbation of COPD or clinical signs of an acute upper respiratory tract infection or had received systemic glucocorticoid therapy 1 month before the surgery. At the same, patients were divided into following two groups based on a smoking history: nonsmokers and smokers. Patients who reported smoking more than 100 cigarettes during their lifetime were defined as smokers. Smoking history was calculated in pack-years as the product of tobacco use (in years) and the average number of cigarettes smoked per day divided by 20 (years x cigarettes per day/20). Pulmonary function was tested by using a pneumotachometric spirometer “CustovitM” (Custo Med, Germany). The highest value of forced expiratory volume in 1 s (FEV_1_), forced vital capacity (FVC) and FEV_1_/FVC ratio from three reproducible measurements were recorded. The results were compared with the predicted value matched for age, body height and sex according to the standard methodology [24].

The study protocol was approved by Kaunas Regional Ethics Committee for Biomedical Research (No. BE-2-20). The study was registered in the U.S. National Institutes of Health trial registry *ClinicalTrials.gov* with identifier NCT02214303.

Written informed consent was obtained from all study subjects.

### Immunohistochemical analysis

**Fig. 1:**
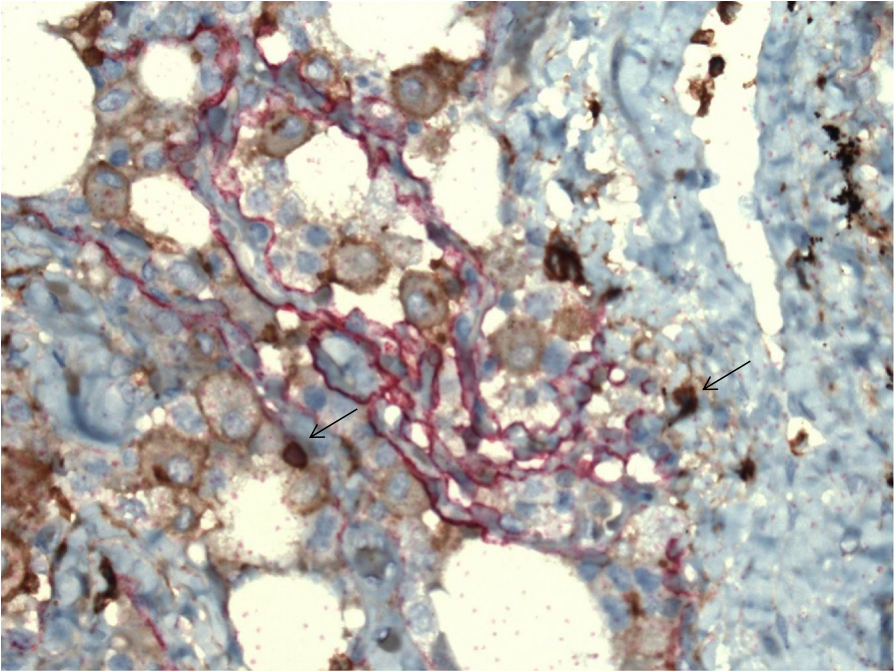
Foxp3^+^CD4^+^ T cells in non-small cell lung cancer tissue. Arrows indicate positive double stained cells. Original magnification: ×400

**Fig. 2:**
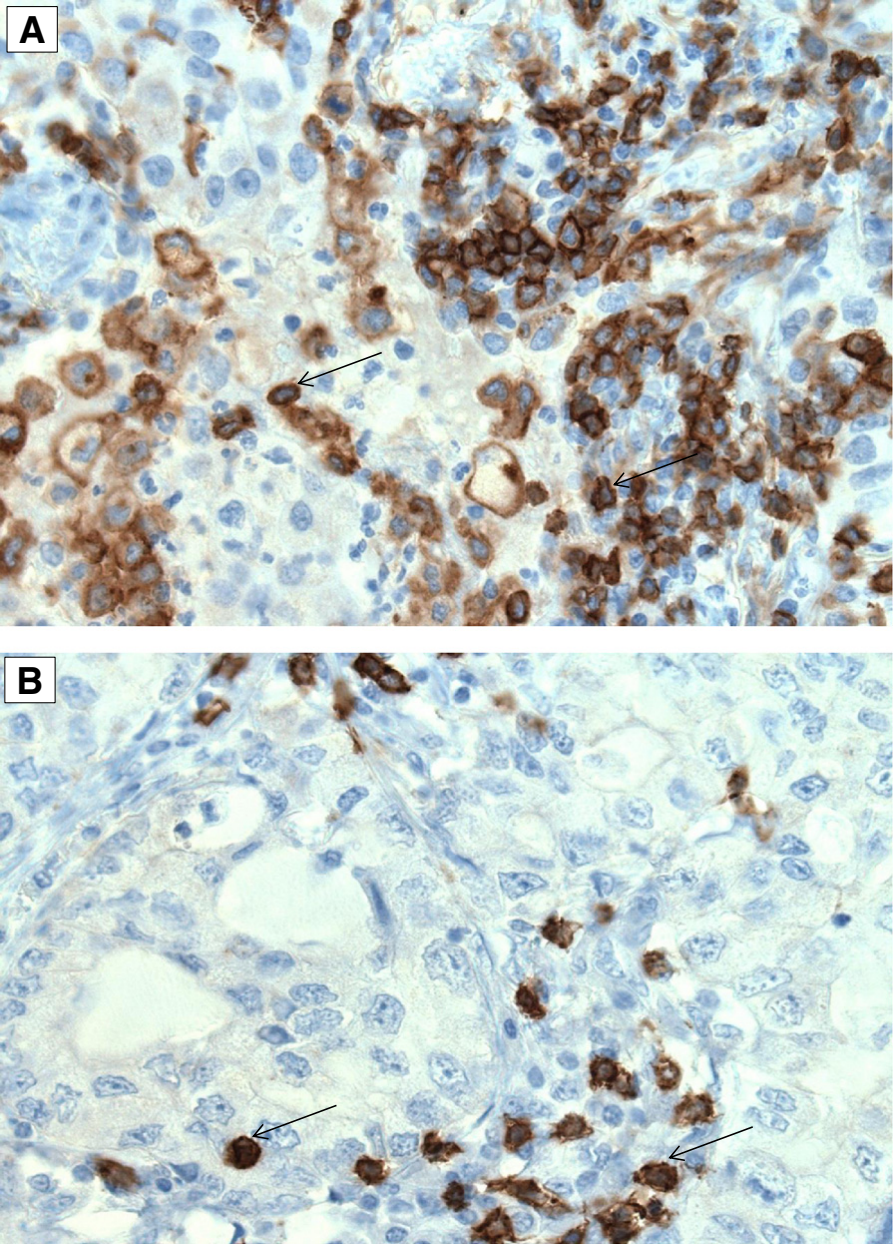
CD4^+^ and CD8^+^ T cells in non-small cell lung cancer tissue. Arrows indicate CD4^+^ (**a**) and CD8^+^ (**b**) positive stained cells. Original magnification: ×400

Lung tissue samples were fixed in formalin and, after dehydration, embedded in paraffin. Tissue sections 3- to 5-μm thick were cut, subsequently de-waxed and rehydrated through graded alcohols. Slides immunohistochemically analyzed for the expression of Foxp3^+^CD4^+^, CD4^+^ and CD8^+^ T cells. A Roche Ventana Benchmark XT automated slide stainer (Ventana Medical Systems, Roche, France) was used for immunohistochemistry. Immunohistochemical staining was performed according to the manufacturer’s instructions. Monoclonal rabbit anti-human antibodies were used for identification CD4^+^ T cells (anti-CD4, SP35, Ventana) and CD8^+^ T cells (anti-CD8, SP57, Ventana). Immunohistochemical double staining was performed to identify Foxp3^+^CD4^+^ T cells using monoclonal rabbit anti-human antibodies against the following proteins: CD4 (anti-CD4, SP35, Ventana), Foxp3 (anti-Foxp3, SP97, Spring). Quantitative evaluation of CD4^+^ and CD8^+^ T cells was done in 10 most representative high-power fields (HPFs × 400 magnification) per tissue section using an Olympus BX50 microscope (Olympus Co, Japan). The number of cells with positive staining was counted manually in two locations: tumor stroma and tumor islets (Figs. 1 and 2). Slides were coded, and microscopic analysis was carried out blindly to the clinical data.

### Peripheral blood collection and detection of cytokine in serum

Peripherial blood samples from all tested patients were collected into sterile vacutainers without additives (2 × 5 mL) and stored at room temperature for the surface clot formation (about 30 min). Then tubes were centrifuged at 1000 × g for 10 min at room temperature. From the upper layer of the sample the serum was vacuumed into sterile cold-resistant Eppendorf tubes and stored at −70 °C for further ELISA analysis.

The serum cytokine levels were measured by enzyme-linked immunosorbent assay (ELISA) according to the manufacturer’s instructions. The minimum detectable concentration of IL-10 was 3.57 pg/mL (IBL International, USA).

### Statistical analysis

All statistical analyses were performed using the Statistical Package for the Social Sciences (SPSS), version 20.0. The number of Foxp3^+^CD4^+^, CD4^+^ and CD8^+^ T cells is presented as median with ranges. The associations between tumor-infiltrating Foxp3^+^CD4^+^, CD4^+^ and CD8^+^ T cells and clinicopathologic characteristics were analyzed using the chi-square (*χ*^2^) test. Differences among all study groups were evaluated by using the Kruskal-Wallis test or the Mann–Whitney *U* test for independent samples and the Wilcoxon test for related samples. Multivariate logistic regression analysis was used to identify factors independently associated with the biomarkers of interest. The Kaplan-Meier method with the log-rank test was used to calculate survival rates and differences in survival curves, and *P* values were determined by the log-rank test. *P* values of <0.05 were considered to indicate statistical significance.

## Results

### Characteristics of studied subjects

Demographic, clinical, and histological characteristics of studied subjects are shown in Table 1. The groups of study patients were homogenous, except for age and smoking pack-years. Our study included 38 patients with adenocarcinoma, 36 patients with squamous cell carcinoma, 5 patients with large cell carcinoma, and 1 patient with adenosquamous carcinoma (the latter two were grouped in other histological group).

**Table 1:** Patients characteristics

Variable	NSCLC	Healthy subjects
	*n* = 80	*n* = 16
Gender n (%):		
Female	16 (20)	3 (18.8)
Male	64 (80)	13 (81.2)
Age, years, median (range)	66 (45–77)	34 (19–77)**
Smoking history n (%)*		
Non- smokers	14 (17.5)	10 (62.5)
Smokers	66 (82.5)	6 (37.5)
Smoking pack-years, median (range)	30 (0–60)	2,5 (0–50)**
FEV_1_ % of pred. median (range)	83 (33–144)	93 (70–114)
FEV_1_/FVC ratio % of pred., median (range)	89 (49–123)	107 (86–114)
Histological NSCLC type:		
Adenocarcinoma	38 (47.5)	_
Squamous cell carcinoma	36 (45)	
Large cell carcinoma	5 (6.2)	
Adenosquamous carcinoma	1 (1.2)	
NSCLC stage:		
Stage IA	4 (5.0)	_
Stage IB	19 (23.8)	
Stage IIA	21 (26.2)	
Stage IIB	5 (6.2)	
Stage IIIA	28 (35.0)	
Stage IIIB	3 (3.8)	
T status:		
T1a	2 (2.5)	_
T1b	11 (13.8)	
T2a	37 (46.2)	
T2b	8 (10.0)	
T3	18 (22.5)	
T4	2 (5.0)	
N status		_
N0	35 (43.8)	
N1	26 (32.5)	
N2	17 (21.2)	
N3	2 (2.5)	
Diferenciation:		_
Poor	46 (57.5)	
Other	33 (41.2)	

*FEV*_*1*_ forced expiratory volume in one second, *FVC* forced vital capacity

*P < 0.05, chi-square (*χ* ^2^) test

**P < 0.05, Mann–Whitney *U* test

### Foxp3^+^CD4^+^T cells infiltration in NSCLC and control group patients

**Fig. 3:**
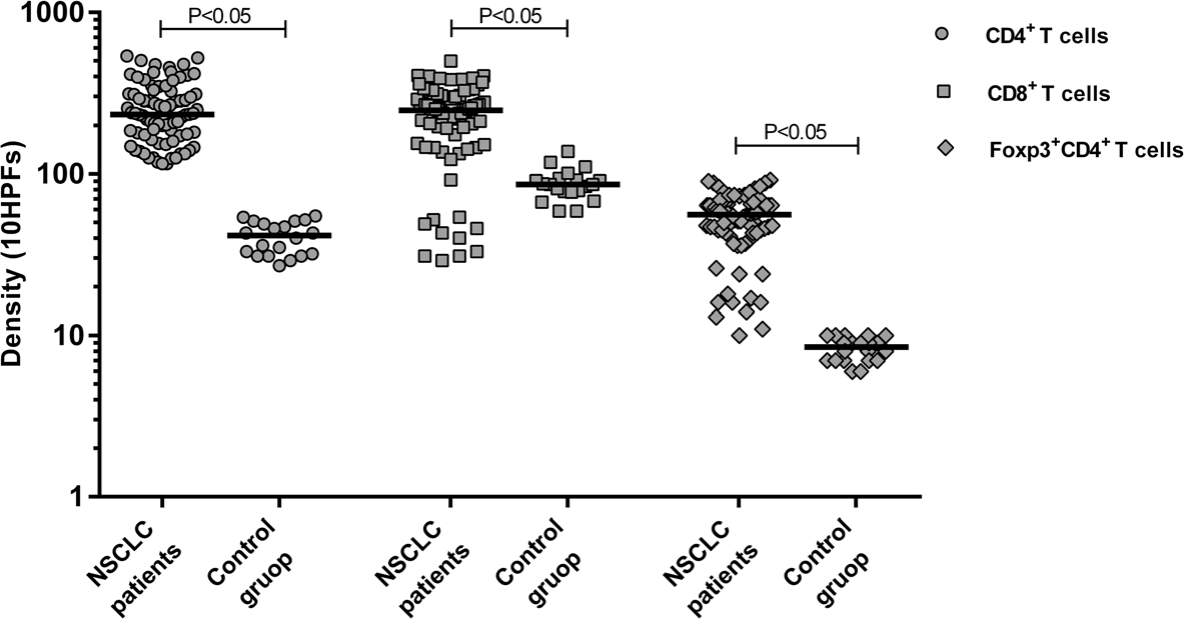
Distribution of total CD4^+^, CD8^+^ and Foxp3^+^CD4^+^ T cells in lung tissue of NSCLC and control group subjects

While analyzing the NSCLC and control group patients, we compared only total numbers of Foxp3^+^CD4^+^ due to different morphological structure of tissue. NSCLC patients had a greater number of lung tissue-infiltrating Foxp3^+^CD4^+^ T cells compared with the control group (*P* < 0.001) (Fig. 3).

### Foxp3^+^CD4^+^ T cells infiltration in tumor islets and stroma

Tumor-infiltrating Foxp3^+^CD4^+^ T cells were observed in the tumor stroma as well as tumor islets with predominant infiltration in the tumor stroma (Table 3). There was no association between the numbers of Foxp3^+^CD4^+^ T cells in tumor stroma or islets and NSCLC patients’ gender, age, pathological T status, lymph node status, histological tumor type, tumor differentiation and smoking or COPD status (Tables 2 and 3).

**Table 2:** Association between total CD4^+^, CD8^+^ and Foxp3^+^CD4^+^ T cells and clinocopathological characteristics

	Median, (range) CD4^+^ T cells	P	Median, (range) CD8^+^ T cells	P	Median, (range) Foxp3^+^CD4^+^ T cells	P
Gender:						
Female	164 (29–456)	NS	195 (68–406)	NS	43 (7–88)	NS
Male	209 (27–538)		244 (59–499)		48 (6–92)	
Smoking status:						
Smokers	116 (29–265)	<0.05	138 (59–332)	<0.05	11 (6–77)	NS
Non-smokers	238 (27–538)		264 (67–499)		52 (6–92)	
Histological NSCLC type:						
Adenocarcinoma	215.5 (116–538)	<0.05 <0.05*	284.5 (134–405)	NS	56.5 (11–88)	NS
Squamous cell carcinoma	282.5 (119–522)		268.5 (109–499)		57 (16–92)	
Other	176.5 (116–417)		288 (92–361)		45 (13–73)	
pT status						
pT1a-2b	232 (116–538)	NS	251 (92–406)	NS	57 (11–92)	NS
pT3-4	238 (116–474)		268.5 (137–499)		53 (16–90)	
Lymph node status:						
Negative (N0)	234 (125–522)	NS	239 (109–403)	NS	57 (11–90)	NS
Positive (N1-N3)	235 (116–538)		270 (92–499)		55 (13–92)	
Diferenciation:						
Poor	253 (116–538)	NS	257.5 (109–499)	NS	56 (11–92)	NS
Other	212 (116–504)		253 (92–406)		58 (13–88)	
COPD:						
Present	222.5 (116–538)	NS	273 (123–405)	NS	56 (16–88)	NS
Absent	282 (126–522)		241.5 (92–499)		56.5 (11–92)	

*P < 0.05, Mann–Whitney test for difference between adenocarcinoma and squamous cell carcinoma

**Table 3:** Association between CD4^+^, CD8^+^ and Foxp3^+^CD4^+^ T cells number within cancer islets and stroma and clinicopathological charateristics

	CD4^+^ T cells median, (range)		CD8^+^ T cells median, (range)		Foxp3^+^CD4^+^ T cells median, (range)	
	Islets	Stroma	Islets	Stroma	Islets	Stroma
Gender:						
Female	27 (11–74)	162 (99–421)	35.5 (16–138)	181.5 (76–319)	12.5 (0–27)	44.5 (11–69)
Male	18 (11–82)	222 (98–489)	39 (10–219)	218 (80–374)	12 (1–35)	41 (12–72)
Smoking status:						
Smokers	18 (11–82)	239 (98–489)**	38 (10–219)	223 (80–374)**	11.5 (0–27)	42.5 (11–63)
Non-smokers	34 (11–74)	138.5 (99–221)	34.5 (16–111)	162 (76–290)	12 (1–35)	41 (12–72)
Histological NSCLC type:						
Adenocarcinoma	17.5 (11–80)	187 (98–458)*,**	39 (10–151)	201.5 (111–374)	11.5 (0–27)	41 (11–69)
Squamous cell carcinoma	18.5 (11–82)	261 (105–489)	36.5 (15–219)	226 (80–362)	13 (3–35)	41 (13–72)
Other	42 (15–57)	127.5 (101–392)	39.5 (16–86)	241 (76–324)	11.5 (1–24)	30.5 (12–63)
pT status						
pT1a-2b	19.5 (11–82)	209 (101–489)	38 (15–151)	206 (76–369)	12.5 (0–32)	44 (11–72)
pT3-4	19.5 (12–69)	213 (98–453)	38 (10–219)	225.5 (115–374)	11.5 (1–35)	40 (13–63)
Lymph node status:						
Negative (N0)	22 (11–78)	215 (98–444)	39 (10–142)	189 (80–374)**	11 (0–35)	42 (11–63)
Positive (N1-N3)	17 (12–82)	205 (99–489)	37 (15–219)	229 (76–369)	13 (1–32)	41 (12–72)
Diferenciation:						
Poor	19.5 (11–82)	223 (98–458)	35.5 (10–219)	207.5 (80–374)	13 (0–35)	40 (11–69)
Other	18 (11–69)	189 (101–489)	39 (15–111)	210 (76–324)	12 (1–25)	43 (12–72)
COPD:						
Present	21.5 (11–80)	191.5 (98–458)	40 (16–219)**	196 (76–374)**	12 (0–35)	40.5 (11–63)
Absent	17 (11–82)	266 (109–489)	34.5 (10–75)	240.5 (101–369)	12 (3–25)	42.5 (13–72)

*P < 0.05, Kruskal-Wallis test

**P < 0.05, Mann–Whitney test

In addition, there were significant correlations between the total number of CD4^+^ T cells and Foxp3^+^CD4^+^ T cells in islets (*r* = 0.26; *P* < 0.05). We found correlations between CD4^+^ T cells in islets and Foxp3^+^CD4^+^ T cells in islets (*r* = 0.58; *P* < 0.05), Foxp3^+^CD4^+^ T cells in stroma (*r* = 0.25; *P* < 0.05) and total Foxp3+/CD4 T cells (*r* = 0.44; *P* < 0.001).

### Foxp3^+^CD4^+^ T cells infiltration and survival in NSCLC

Better survival was found for NSCLC patents with higher Foxp3^+^CD4^+^ T cells infiltration in tumor islets, stroma or higher total Foxp3^+^CD4^+^ T cells infiltration (*P* < 0.05). Since previous studies have analyzed ratios between Foxp3^+^CD4^+^ and CD8^+^ T cells in relation to survival, we also calculated the associations of the CD8^+^/Foxp3^+^CD4^+^ ratio with survival. Patients with higher CD8^+^/Foxp3^+^CD4^+^ ratio were associated with better overall survival (*P* < 0.05).

### CD4^+^ and CD8^+^ T cells infiltration in NSCLC and control group patients

NSCLC patients had a greater number of lung tissue-infiltrating CD4^+^ and CD8^+^ T cells compared with the control group (*P* < 0.001) (Fig. 3).

### CD4^+^ and CD8^+^ T cells infiltration in tumor islets and stroma

Predominant infiltration of CD4^+^ T cells as well as CD8^+^ T cells was observed in tumor stroma compared to tumor islets (*P* < 0.001). CD8^+^ T cells predominated over CD4^+^ T cells in the tumor islets (*P* < 0.001), but no significant difference between the numbers of these cells was found in the tumor stroma. There was no association between the numbers of CD4^+^ and CD8^+^ T cells in tumor stroma or islets and NSCLC patients’ gender, age, pathological T status.

Smoking patients with NSCLC had a significantly greater number of tumor stroma-infiltrating CD4^+^ T cells than nonsmokers with NSCLC (*P* < 0.05). Also greater number of CD8^+^ T cells in cancer stroma and total tumor infiltrating CD8^+^ T cells was found in smoking NSCLC patients compared to non-smokers NSCLC patients (*P* < 0.05) (Table 3).

More CD8^+^ T cells in tumor stroma were presented in NSCLC with COPD comparing with NSCLC without COPD patients, and contrary, more CD8^+^ T cells in tumor islets were found in NSCLC patients without COPD compared to NSCLC patients with COPD (*P* < 0.05) (Table 3).

While analyzing the total number of CD4^+^ T cells, they were more frequently found in squamous cell carcinoma than adenocarcinoma (*P* < 0.05) (Table 2). CD4^+^ T cells were found more abundantly in the stroma of squamous cell carcinoma compared with adenocarcinoma (*P* < 0.05) (Table 3).

Also greater number of CD8^+^ T cells in cancer stroma was found in NSCLC patients with lymph node metastases compared to NSCLC patients without lymph node metastases (*P* < 0.05) (Table 3).

In addition, there were significant correlations between the total number of CD4^+^ T cells and CD8^+^ T cells in islets (*r* = 0.39; *P* < 0.001) as well as in stroma (*r* = 0.36; *P* < 0.05) and total CD8^+^ T cells (*r* = 0.47; *P* < 0.001). CD4^+^ T cells in stroma correlated with CD8^+^ T cells in islets (*r* = 0.36; *P* < 0.05) in stroma (*r* = 0.36; *P* < 0.05) and in total (*r* = 0.46; *P* < 0.001).

### CD4^+^ and CD8^+^ T cells infiltration and survival in NSCLC

It was found that CD4^+^ and CD8^+^ T cells were not associated with overall survival. Patients with higher CD4^+^/CD8^+^ ratio were associated with better overall survival (*P* < 0.05).

### IL-10 levels in serum

**Fig. 4:**
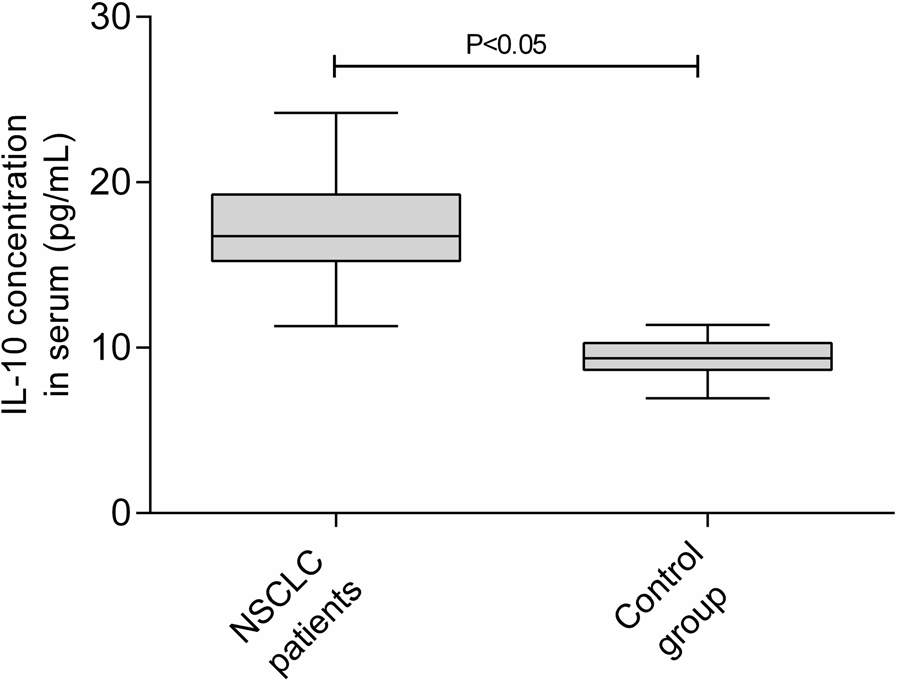
Serum IL-10 concentration in NSCLC and control group patients

We examined serum IL-10 in NSCLC patients and compared with taht in control group patients. NSCLC patient had remarkably higher levels of serum IL-10 concentration (Fig. 4).

Also we investigated correlations between tumor infiltrating T cells and serum IL-10. We found correlation between total CD4^+^ T cells count and IL-10 (*r* = 0.67; *P* < 0.001). CD4^+^ T cells in stroma correlated with IL-10 (*r* = 0.66; *P* < 0.001). CD8^+^ T cells in islets correlated with IL-10 (*r* = 0.28; *P* < 0.05).

To investigate the association between overall survival of NSCLC patients and immune cell infiltration, multivariate logistic regression analysis was performed. A high level of Foxp3^+^CD4^+^ T cells infiltration in the tumor stroma was found to be independently associated with the better overall survival (Exp(B), 3.69; 95 % CI of Exp(B), 1.05–12.96; *P* = 0.041).

## Discussion

The goal of our study was to examine tumor infiltrating Foxp3^+^CD4^+^, CD4^+^ and CD8^+^ T cells by immunohistochemistry in specimens of NSCLC patients and evaluate the prognostic value of these cells especially of Foxp3^+^CD4^+^ .

In agreement with earlier reports, the current study confirmed the the importance of CD8^+^, CD4^+^ and Foxp3^+^CD4^+^ T cells as prognostic factors. The novelty of our study was that these cells were examined in both normal and malignant tissues.

Differently from normal tissue, lung tumors have two distinct but interdependent compartments: the parenchyma (neoplastic cells) and the stroma that the neoplastic cells induce and in which they are dispersed, that is why while analyzing control and NSCLC groups we used total number (in tumor islets and stroma) of Foxp3^+^CD4^+^, CD4^+^ and CD8^+^ T cells. As expected, the numbers of Foxp3^+^CD4^+^, CD4^+^ and CD8^+^ T cells were greater in the NSCLC tissue than the lung tissue from control group subjects. To our knowledge, there are no studies that compared T cell infiltration in the lung tissue between lung cancer patients and control subjects.

Foxp3^+^CD4^+^ T cells are generally considered to be immunosuppressive and have been linked to poor outcome in several types of solid tumor also in NSCLC. The major original findings of our study were that they are associated with better survival.

Contrary to the putative pro-tumorigenic effect, the presence of Foxp3^+^CD4^+^ T cells has been associated with a good prognosis in colorectal and head and neck cancers [25–27]. FOXP3 expression has been recently described in normal cells and in non-hematopoietic-derived cancer cells, suggesting that FOXP3 biological effects are not restricted to Foxp3^+^CD4^+^ T cells. FOXP3 expression has been observed in different cancer histotypes (NSCLC, breast, urinary bladder, tongue, gastric, esophageal, pancreas, colorectal, stomach, thyroid, glioma and melanoma). Tumor infiltration by Foxp3^+^CD4^+^T cells, near-to-invariably detected by the immunohistochemical detection of Foxp3^+^ lymphocytes, has been associated with poor prognosis in cohorts of patients affected by multiple distinct neoplasms, includ-ing Hodgkin lymphoma, melanoma as well as breast, gastric and ovarian carcinoma [25, 28–31].

Some authors reported good prognostic value of Foxp3^+^CD4^+^ T cells in head and neck cancer [32, 33], colorectal carcinoma [26, 34] bladder cancer [35] and lymphoma [36]. In some tumor types including hepatocellular carcinoma and breast cancer, high levels of intratumoral Foxp3^+^CD4^+^ T cells have been associated with bad prognosis [37]. The discrepancy could arise from the differences in methodologies or biological properties of cancers. The FoxP3 was originally known as a nuclear marker of CD4^+^CD25^+^ regulatory cells, but compelling data have showed that it can also be expressed in the effector or other helper T cells [38, 39] indicating that apart from Foxp3^+^CD4^+^ T cells, FoxP3^+^ cells incorporate several other subgroups. Ronald J reviewed 58 studies including 16 different malignant tumors and found that the prognostic value of FoxP3 varies widely even in the same cancer type. Nevertheless, in four of these studies in which double labeling of FoxP3 combined with CD4^+^, CD25^+^ or CD8^+^ was applied, the double-positive T cells were generally associated with a poor prognosis except colorectal cancer [37]. These findings suggest that Foxp3^+^CD4^+^ T cells might play a dual role in carcinogenesis.

Various factors may explain these seemingly contradictory observations. Intratumoral Foxp3^+^CD4^+^ T cells have been shown to display a consistent degree of functional heterogeneity, which may obviously influence prognosis. Also distinct subsets of Foxp3^+^CD4^+^ T cells appear to specifically control limited subsets of T cells [40]. Some cancers, such a lymphoma, head and neck cancer, gastric cancer and colorectal carcinoma has an important proinflammatory component. Therefore Foxp3^+^CD4^+^ T cells mediated immunosuppression may exert antitumor functions. Some studies showed that the ratio between tumor infiltrating CD8^+^T cells and Foxp3^+^CD4^+^ T cells gives prognostic/predictive information than either parameter alone. In particular, a high CD8^+^/Foxp3^+^CD4^+^ T cells ratio has been associated with favorable disease outcome in ovarian and hepatocellular carcinoma patients [30, 41] and with a poor prognosis in subjects affected by head and neck cancer [42]. Our study results showed that a high CD8^+^/Foxp3^+^CD4^+^ T cells ratio has been associated with favorable disease outcome (*P* < 0.05), meanwhile CD4^+^ or CD8^+^ T cells counts separately didn’t significantly afected disease outcome.

Foxp3^+^CD4^+^ T cells may benefit the host by modulating inflammation, which is linked to the angiogenesis of cancer [14].

Foxp3^+^CD4^+^ T cells inhibit the activation of both CD4^+^ and CD8^+^ T cells, and within the tumor microenvironment may serve to suppress anti-cancer cell immunity. In some murine tumors (melanoma and fibrosarcoma), both CD4^+^ and CD8^+^ T cells were found to be required for tumor rejection. Since CD4^+^ and CD8^+^ T cells has been described as a positive prognostic factor in NSCLC [43–45], we performed an analysis of CD4^+^ and CD8^+^ T cells in relation to disease survival. Tumor infiltrating CD4^+^, CD8^+^ T cells were associated with neither overall survival nor disease-free survival. Contrasted with the possible tumor-promoting role of these cells in the progression of established tumors, the role of Foxp3^+^CD4^+^ T cells in initial tumor transformation caused by chronic inflammation remains elusive. Based on the potent immunoregulatory function these cells have on immune responses and inflammation, it is reasonable to conceive that Foxp3^+^CD4^+^ T cells may in fact help prevent or delay inflammation-mediated tumor development. However, the experimental evidence supporting this notion awaits further investigation [46].

There are indications that IL-10 is reduced in patients with NSCLC and that this reduction could correlate with poor prognosis [11, 12]. However, up to now a connection between higher levels of IL-10 and better survival could not been shown [47]. We did not find any associations between IL-10 and NSCLC patients outcomes.

## Conclusions

Our study demonstrated that tumor infiltrating Foxp3^+^CD4^+^T cells are associated with improved NSCLC patients’ survival. In addition our findings highlight a tendency of high CD4^+^/CD8^+^ and CD8^+^/Foxp3^+^CD4^+^ T cells ratio in prolonged NSCLC patients’ survival.
